# Safe long-term therapy of Cushing’s syndrome over 37 years with mitotane

**DOI:** 10.3389/fendo.2024.1294415

**Published:** 2024-02-19

**Authors:** Jonas Seibold, Mario Hönemann, Anke Tönjes, Benjamin Sandner

**Affiliations:** ^1^ Medical Department III - Endocrinology, Nephrology, Rheumatology, University of Leipzig Medical Center, Leipzig, Germany; ^2^ Institute of Medical Microbiology and Virology, University of Leipzig Medical Center, Leipzig, Germany

**Keywords:** mitotane, Cushing’s syndrome, pregnancy, long-term treatment, adrenostatic drug

## Abstract

While suggested, surgery is not always possible as a first-line treatment of Cushing’s Disease (CD). In such cases, patients require medical therapy in order to prevent complications resulting from hypercortisolism. Although there has been a wide expansion in pharmacological options in recent years, mitotane was the agent of choice for treating hypercortisolism decades ago. Due to the introduction of other therapies, long-term experience with mitotane remains limited. Here, we report the case of a woman with CD who was treated with mitotane for 37 years. During the treatment period, biochemical and clinical disease control was achieved and the patient had two uncomplicated pregnancies. Drug-related side effects remained moderate and could be controlled by several dose adjustments. Our case highlights the ability of mitotane to allow an effective control of hypercortisolism and to represent a safe treatment option in special situations where CD requires an alternative therapeutic approach. Furthermore, we provide a literature review of the long-term use of mitotane and reported cases of pregnancy in the context of mitotane therapy.

## Introduction

The majority of cases of endogenous hypercortisolism (*Cushing’s syndrome*) occur due to an ACTH (adrenocorticotropic hormone) producing adenoma of the pituitary gland (so-called *Cushing’s disease (CD)*) (1).

For all etiologies of Cushing’s Syndrome, resection of the causal lesion(s) represents the treatment of choice (2). However, certain situations may require an alternative therapeutic approach. Medical therapy can be used as a primary or adjunctive option if the primary lesion is not fully resectable (e.g., due to size/location of the tumor or metastatic disease), if a surgical approach is not feasible due to severe comorbidities, or in line with the patient’s preference (1, 3).

Furthermore, drugs may help to control hypercortisolism in patients who suffer acute complications by severe hypercortisolism as well as in situations with persistent or recurrent symptoms after pituitary surgery, which may be the case in up to 20% of patients (4).

Various medical agents with different points of action have been identified for the treatment of hypercortisolism. Pituitary-directed drugs (e.g. pasireotide, cabergolin) inhibit central ACTH-secretion, whereas adrenal-directed drugs (e.g. ketoconazole, metyrapone) directly disrupt adrenal steroidogenesis. Recently, several new steroidogenesis inhibitors have been under investigation. Osilodrostat and levoketoconazole (the 2S, 4R enantiomer of ketoconazole), whose efficacy has now been demonstrated in several studies, offer certain advantages such as improved pharmacokinetics and reduced toxicity (3, 5, 6). Glucocorticoid receptor antagonists do not alter hormone production but inhibit the glucocorticoid receptor (7).

*Mitotane* (o,p’-dichlorodiphenyldichloroethane, o,p’-DDD), a derivate of the insecticide DDT (dichlorodiphenyltrichloroethane) (8), exhibits both adrenolytic and adrenostatic effects (9–11). Discovered in the 1950s, its cytotoxic effects were soon utilized for the treatment of adrenocortical carcinoma, where it showed beneficial effects on survival and long-term remission (12–14). Though not fully understood, the cytotoxic effects are thought to be mediated by the inhibition of mitochondrial respiratory chain complexes and the induction of endoplasmic reticulum (ER) stress. Subsequently, this leads to cell apoptosis and impairment of overall steroidogenesis with an almost mandatory necessity of glucocorticoid replacement therapy (11, 15–18). In contrast, mineralocorticoid deficiency is less frequent, as glomerulosa cells appear to be less affected by mitotane. Therefore, fludrocortisone replacement therapy was commenced in only 20 – 65% of patients usually due to orthostatic hypotension or dizziness. In addition, mineralocorticoid insufficiency occurred later, typically 6 – 9 months after initiation of mitotane (10, 19, 20).

At lower concentrations, mitotane predominantly inhibits several steps in adrenal steroid synthesis (e.g., cholesterol side-chain cleavage enzyme and 11-beta-hydroxylase), whereas cytolytic effects are reduced (9, 11, 19). Moreover, by an enhanced cortisol clearance through induction of cytochrome P450 3A4 (CYP3A4), and through increased levels of corticosteroid-binding globulin (CBG, transcortin), mitotane reduces the levels of biologically active cortisol (18, 20–22).

Hence, it can be used as an adrenostatic agent in ACTH-dependent hypercortisolism, where it has not only proven to be one of the most effective drugs in normalizing cortisol levels but also to have beneficial effects on clinical features, even in long-term treatment (7, 23–25). However, experience with mitotane in the treatment of CD is limited, especially regarding long-term treatment and its use under special circumstances.

## Case history

The patient first presented as a 9-year-old girl at Leipzig University Hospital with cushingoid habitus, acne, and hirsutism in 1969. She was overweight and below average height for her age. Elevated levels of 17-ketosteroids and hydroxycorticosteroids were found in the patient’s urine, whereas imaging of the adrenal glands and the pituitary region (aortography of the adrenal glands and sella x-ray) showed no pathological findings. Clinical symptoms worsened throughout the next visits, in particular persistent growth retardation and development of a buffalo hump. Furthermore, an impaired glucose tolerance developed. Based on the loss of the physiological circadian rhythm of cortisol serum levels and a lack of suppression of cortisol in the 1 mg dexamethasone suppression test (DST), the diagnosis of endogenous hypercortisolism was established. Since ACTH assays as well as CT or MRI scans were not available at the hospital at that time, no further diagnostic workup was possible. Due to her religious beliefs (being Jehovah’s Witnesses), the patient’s mother decided against the recommended adrenalectomy, and the patient was discharged without any treatment.

At 16 years of age, as an operative treatment was still strictly rejected by the patient’s parents, medication with mitotane was initiated at a loading dose of 2 grams per day. At that time the patient weighed 65 kilograms, resulting in a weight-for-age above the 75^th^ percentile. Within 3 months she lost 8 kilograms in weight and showed a normalisation of steroid levels. Mitotane treatment was monitored by periodical evaluation of the plasmatic concentration. The drug, by then downscaled to 1 gram per day, was well tolerated and, with the exception of elevated transaminases, no biochemical abnormalities were found. Within 12 months, the symptoms of hypercortisolism improved, her weight dropped to 47 kilograms, and a normal menstrual cycle developed. Laboratory analyses confirmed a normalization of transaminases and glucose metabolism.

Over the next years, further therapy was conducted in an outpatient setting closer to the patient’s hometown. During the first months, two hospitalizations became necessary due to gastroenteritis and malaise with vertigo, requiring short-term discontinuation of the drug with a subsequent dose adjustment. However, no overt signs of adrenal insufficiency were reported.

At the age of 23, with a daily dose of 750 mg mitotane, the patient gave birth to a boy via an uncomplicated primary cesarean delivery in the 35^th^ week of gestation. Neither the mother nor the boy showed signs of adrenal insufficiency. Perioperatively, the mother was given prednisolone as a substitute. In response to slightly elevated serum cortisol levels after discharge, mitotane was titrated to the personal maximum dose of 1250 mg daily. At age 31 and still on the same amount of mitotane, the patient gave birth to another healthy boy. Both boys developed normally during infancy and adolescence and showed no signs of adrenal insufficiency to this day (being 40 and 32 years old at the time of this article).

At the age of 36, new features of hypercortisolism developed under 500 mg of mitotane per day. Further diagnostic workup for CD showed a lack of suppression in the 1 mg DST with elevated ACTH levels and a CRH stimulation test with a typical increase in ACTH and cortisol levels. As pituitary MRI showed no adenoma, petrosal sinus sampling was performed and excluded the ectopic origin of hypercortisolism (pituitary/peripheral gradient for ACTH was 35-fold on the left and 15-fold on the right). Subsequently, the patient consented to the proposed transsphenoidal surgery. Similarly to previous findings, no adenoma could be detected intraoperatively, and a central 1/3 resection of the pituitary was carried out. The histological examination showed discrete hyperplasia of ACTH-producing cells. As the symptoms of CD persisted, mitotane was continued. Even after a later hospitalization with sepsis in the context of massively elevated mitotane levels, the patient decided against radiation of the pituitary, as well as alternative medication with pasireotide, but insisted on continuing to take mitotane on her own responsibility. Over the long period of treatment serum concentrations of mitotane were very regularly monitored and consistently found within the target range.

In early 2013, the patient suffered a right-sided middle cerebral artery (MCA) stroke, and just a few months later, she was hospitalized due to an acute adrenal insufficiency in the context of an urosepsis. In the same year, she was admitted to our intensive care unit with an acute adrenal insufficiency and a massive exsiccosis. Clearly reduced morning serum cortisol levels (24 nmol/l) and a subsequently performed ACTH stimulation test revealed a primary adrenal insufficiency (reference range (RR) > 500 nmol/l). Replacement therapy with hydrocortisone and fludrocortisone was established due to a concomitant mineralocorticoid deficiency, whereas mitotane was stopped at a plasma level of 18.6 mg/l. Follow-up blood samples taken two months after discharge showed persistent primary adrenal insufficiency.

When the patient once again presented with symptoms of hypercortisolism in 2018, midnight cortisol levels were within normal ranges (42 nmol/l, ULN < 207). Although daytime cortisol values showed a circadian dynamic, the basal ACTH on the other hand was elevated to 31.45 pmol/l (RR 1.04 – 10.8). As the DST and brain MRI findings were unremarkable, the symptoms were not considered to require a specific treatment, even though the continuously elevated ACTH levels may suggest the persistence of central hypercortisolism. Since then, there have been regular endocrinological diagnostic work-ups, including regular MRI examinations, and the clinical situation remained unaltered. Moreover, no severe medical conditions have developed thus far.

## Discussion

Mitotane is the first-line treatment for adrenal carcinoma. One of its complex modes of action is its adrenolytic effect, resulting in a reduction of cortisol levels. Therefore, it presents one second-line treatment option also for CD. However, data from long-term applications is missing (2). A review of the literature revealed less than 200 reported cases with long-term use of mitotane (**Table 1**), with the longest reported intake period being 11.5 years (26).

**Table 1:** Published case reports of long-term treatment with mitotane.

Study	Entity	Patients [*n*]	Treatment duration^1^	Follow-up^1^	Dose [mg/d]	Mitotane plasma concentration [mg/l]	AI After treatment	Other side effects
Knappe et al., 1997 (26)	CD	6	(1,5-)11,5y	11,5 y	max. 3000	*n/a*	17% (1) (after 5 y)	↑ GGT, ASAT, ALAT, diarrhea (in 1 patient)
Sharma et al., 2012 (27)	EAS	1	1,5 y	3 y	500-2000	*n/a*	100% (1) hypercortisolism recurred 16 m later	*n/a*
Kamenický et al., 2011 (28)	4 CD 7 EAS	11	(1–) 30,5 m median 3.0 m	1-42 m median 14 m	median 3000	median 10,1 (4,3-13,9) combination therapy: median 2,9 (1,9-6,0)	9% (1) permanent AI (after 30,5 m) 36% (4) acute AI	gastrointestinal discomfort, ↑ GGT, ASAT, ALAT, ↑ cholesterol dizziness, confusion Hypokalemia
Baudry et al., 2012 (23)	CD	76	9,5 y median 10,3 m	mean 97 m (6.3–192)	mean 2600 ± 1100	10,5 ± 8.9	75% (57) at withdrawal of Mitotane	gastrointestinal discomfort, ↑ GGT, transaminases, ↑ LDL-, HDL-cholesterol, neutropenia, skin rash, gynecomastia
Luton at al. 1979 (24)	CD	62	3-34 m	*n/a*	4000-12000	*n/a*	31% (19)	*n/a*
Poirier et al., 2020 (29)	ACC	23	(24-) 85 m median 34 m	24 – 144 m	max. 2000-4500	mean peak concentration 19.4 mg/ml* (8.1-27.1 mg/ml)	21,7% (5) permanent AI, others achieved recovery	*n/a*
Keller et al., 1988 (13)	CS	3	(5-) 10y	(5-) 10y	15-20 mg/kg	*n/a*	none	↑ cholesterol
Benecke et al., 1991 (30)	CD	2	5-8 y	(5-) 8 y	375-6000 (maintenance dose ca. 1000)	1-17	none	gastrointestinal discomfort, central nervous depression
Ichikawa et al., 1999 (31)	CD	1	18 y	18 y	1000-4000	*n/a*	none	nausea and vomiting

CD, Cushing’s disease; CS, Cushing’s syndrome; EAS, ectopic ACTH syndrome; ACC, adrenocortical cancer; HC, hypercortisolism; y, years; m, months; AI, adrenal insufficiency; GGT, gamma-glutamyltransferase; ASAT, aspartate aminotransferase; ALAT, alanine aminotransferase; LDL, low-density lipoprotein HDL, high-density lipoprotein; n/a, no data/information available; ↑, rise/elevation.

^1^unless specified otherwise the longest mitotane treatment or follow-up mentioned in the study is listed.

*contrary to other publications the authors report mitotane plasma levels in mg/ml.

To the best of our knowledge, we hereby reported the longest documented treatment using mitotane, with a total duration of 37 years. This exceedingly long exposure allowed an exceptional insight into the potentials and challenges of therapy with mitotane. Contrary to many other reported cases, we saw a patient on exclusive single-drug therapy with mitotane, allowing us to examine the drug’s efficacy and side effects without any interference from other medication.

In our patient, the signs and symptoms of hypercortisolism regressed and a normalization of steroid levels was achieved within months after the initiation of mitotane. As specified by a recent meta-analysis by Broersen et al. (25), mitotane provides an effective control of cortisol levels in 80% of CD patients, and in that respect is the most effective drug among pasireotide, cabergoline, ketoconazole, and metyrapone.

In comparison, our patient’s daily dose of 125 to 1250 mg was lower than what was reported in other studies, e.g., 2600 ± 1100 mg described by Baudry et al. (23). Even though several dose adjustments were made due to side effects, the regimen could be maintained without the need for a dosage escalation over time. This underlines that, contrary to adrenostatic drugs, mitotane does not tend to treatment escape (4).

However, while the adrenolytic effects of mitotane are reduced at lower doses, adrenal insufficiency (AI) occurs regularly. Subsequently, glucocorticoid replacement becomes necessary in most of the patients (**Table 1**) (7, 10). Nonetheless, mitotane therapy without simultaneous steroid replacement was possible in our patient by performing both close clinical and laboratory monitoring.

It may be difficult to maintain the balance between beneficial and adverse drug effects. Mitotane shows high accumulation in adipose tissue, causing a half-life of up to 5 months (32). Differences in the individual intestinal metabolism and a correlation between free mitotane levels, considering the active fraction, and individual cholesterol and triglyceride concentrations fuel variability in drug levels (33). The drug`s toxic effects are hard to predict as they vary considerably and do not necessarily correlate with plasma levels. Furthermore, mitotane has a high potential for drug-drug interactions and an inductive effect on several CYP-enzymes (15). Therefore, side effects occurred in most of the reported patients and made permanent discontinuation of mitotane necessary in one-third of the cases (20, 23).

Over the years, our patient experienced a wide range of the most commonly reported toxic drug effects (**Table 1**), such as elevation of transaminases, gastrointestinal and neurological toxicities, as well as recurrent severe infections, which may indicate immune deficiency (23, 26, 34, 35). However, as the main part of the side effects appears to be dose-dependent and reversible (26, 34), they could be managed by several dose adjustments.

A remarkable aspect of our patient’s history is that she repeatedly developed severe anemia, which resolved within a few weeks after the withdrawal of mitotane. Even if rarely reported, observations from animals exposed to DDT isomers suggest a causal relationship, possibly due to inflammation-associated hypoferremia, inhibition of hematopoietic tissue, or an estrogenic inhibitory effect on red blood cells (15, 36, 37).

Taken together, our case highlights that through close collaboration with well-informed patients and regular evaluations of the dosage, toxic drug effects are manageable and long-term treatment is safe and effective.

The latest follow-up examinations of our patient revealed a physiological cortisol circadian dynamic, suggesting an at least partial, recovery of the adrenal function. Hypothalamus-pituitary-adrenal axis recovery after discontinuation of mitotane was also observed by other authors in up to 80% of their patients with a mean time to recovery of 2.7 years (28, 29). Although no predictors for recovery have been identified so far, this further supports the theory that adrenocortical tissue remains viable and normal adrenal function can be regained with lower drug concentrations (29).

### Fertility during mitotane treatment

One of the unique aspects of the presented case is that the patient gave birth to two healthy children while being treated with mitotane. This is remarkable, as not only hypercortisolism (38, 39) but also exposure to DDT and its isomers may affect fertility and pregnancy outcomes negatively (40–42).

Even though the majority of women with CD suffer from oligo- or amenorrhea (39), the history of our patient points out that mitotane provides sufficient control of cortisol levels. Therefore, a normal menstrual cycle and fertility could be restored.

Furthermore, despite the negative effects of DDT/DDD on gonadal function, which was established in an animal model (43) as well as in humans (20, 34), our case underlines that even at therapeutic levels of mitotane (14 – 20 mg/l), conception is possible (35).

Due to the negative impact of DDT and its derivates on pregnancy and fetal outcomes observed in animal and environmental studies (40, 44, 45), experts recommend avoiding pregnancy when taking mitotane (2). Consequently, the influence of mitotane on fetal development remains poorly understood and it is not clear whether the effects observed in DDT will occur in humans exposed to o,p’-DDD. Overall, we found a total of 21 documented pregnancies in association with mitotane intake (**Table 2**), the majority of which were terminated by therapeutic abortion.

**Table 2:** Published case reports of pregnancy under mitotane intake.

Study	Entity	Pregnancies [*n*]	Dose [mg/d]	Time of mitotane arrest	MMP [mg/l]	Age of the mother	Outcome	Birthweight [g]	Sex of the child	Adrenal function at birth	Appearance of external genitalia	Neurological development
Leiba et al., 1989 (42)	CD	1	*n/a*	week 4	*n/a*	30 y	TA at week 6	*n/a*	*n/a*	dysmorphogenic primordial cortex	*n/a*	*-*
Gerl et al., 1992 (46) *^1^*	CD	1	1000- 1500	until week 34 at 1000 mg/d	pregnancy: 3,9-4,7 delivery: 1,4 cord blood: 1,4	26 y	spontaneous birth at week 38	normal	m	elevated ACTH, normal cortisol, no clinical signs of AI	normal	normal
Knappe et al., 1997 (26) *^1^*	CD	1	n/a	discontinued before	*n/a*	*n/a*	uncomplicated pregnancy	*n/a*	*n/a*	*n/a*	*n/a*	*n/a*
Kojori et al., 2011 (47)	ACC	1	1000	continued	*n/a*	27 y	HELLP syndrome, premature C.s. week 32	1409 (10. pct)	m	APGAR 6, resp. distress, normal ACTH, cortisol	normal	normal
Baszko-Blaszyk et al., 2011 (48)	ACC	1 (twins)	1500	continued	conception: 12,5	26 y	twins spontaneous abortion at week 10	*n/a*	*n/a*	*-*	*n/a*	*-*
Tripto-Shkolnik et al., 2013 (49)	ACC	1	*n/a*	week 6	conception: 9,8 week 21: 5,99 cord blood: 0	33 y	TA at week 21	*n/a*	f	*-*	normal	*-*
de Corbiere et al., 2015 (50)	ACC	1	*n/a*	*n/a*	*n/a*	39 y	TA at week 8	*n/a*	*n/a*	*-*	*n/a*	*-*
Magkou et al., 2018 (51)	ACC, CD	4 (1 twin pregnancy)	2000 – 3000	one discontinued 5 m before, others stopped at week 6-9	conception: 0,9-6,7 term: n.d. - 1,7	20 y, 25 y, 20 y	emergency week 40 (C.s.)*, 40 (C.s.)*, spontaneous at 39 (C.s.), 40 (VD)	2180, 2550, 3470, 4450	f, f, f, m	normal in 1 case DHEA-S decreased	normal	normal
Baudry et al., 2012 (23)	CD	4 (10)	*n/a*	discontinued before conception	*n/a*	*n/a*	TA in the 6/10 of continued mitotane; normal pregnancy in 4/10	*n/a*	*n/a*	*n/a*	normal	*n/a*

ACC, adrenocortical cancer; AI, adrenal insufficiency; CD, Cushing’s disease; C.s., Caesarian section; DHEA-S, dehydroepiandrosterone sulfate; f, female; m, male; MMP, mitotane in maternal plasma; n/a, no data/information available; n.d., not detectable; pctl, percentile; TA, therapeutic abortion; VD, vaginal delivery; y, years.

^1^different pregnancies in the same mother.

Whereas several investigations suggest a negative impact of maternal exposure to DDT isomers on fetal neurologic development (52–54), in the context of mitotane, only Leiba et al. found a nervous system malformation in a fetus after abortion (42). In contrast, our patient’s sons, as well as the other live births, reported in **Table 2**, showed no neurologic impairments. Their normal educational and vocational development thus extends the findings of Magkou et al. (51), showing normal neurocognitive development throughout early childhood after exposure to mitotane in pregnancy.

Acute adrenal insufficiency, due to cytolytic effects on the fetus’ adrenal glands, was neither observed in our patient’s sons nor described in the other cases. This indicates that there is no impact on the offspring’s adrenal glands, or at least the possible impact remains tolerable at the applied doses. However, toxicity at higher plasma levels with an underlying dose-effect relationship, as seen in acute toxicity of mitotane, cannot be ruled out according to the available data.

In the examined studies, continued intake of mitotane was described only for one other case with a premature operative delivery subsequent to the development of a HELLP syndrome (hemolysis, elevated liver enzymes, and low platelet count) (47). In another case, a healthy infant was born after mitotane cessation at the 34^th^ gestational week, with detectable maternal plasma levels at birth (26). In all the other live births, mitotane was either stopped before conception or in the first trimester. There was no regular evaluation of o,p’-DDD-plasma levels throughout pregnancy in most of the presented cases and there are inconsistent findings regarding the extent to which mitotane is transferred from the mother to the fetus (26, 49, 51).

Moreover, due to the small number of natural pregnancies, it is not possible to assess whether the increased risk of premature delivery, spontaneous abortion, or small-for-gestational-age that are associated with exposure to DDT/DDE, are also present in the case of mitotane exposure (45, 55, 56).

In summary, the data on the use of mitotane during pregnancy is incomplete. However there is also very little experience with other adrenostatic medications for pregnant women (2). Mitotane represents a possible therapy for patients for whom surgical therapy is not possible or desirable, also in long-term application.

## Summary/Conclusion

In conclusion, we report the case of the longest documented application of mitotane in a patient with CD. In addition, we highlight the ability of mitotane to provide an efficient control of levels of cortisol, while adverse effects of the drug are manageable. Our patient’s medical history, underlined by the uncomplicated pregnancy and parturition of two healthy children, suggests that mitotane may be a long-term alternative if surgery or other therapeutic options are not feasible.

## Author contributions

JS: Writing – original draft. MH: Writing – original draft, Writing – review & editing. AT: Writing – review & editing. BS: Writing – review & editing.
